# The Royal Infirmary, Dundee

**Published:** 1893-01-07

**Authors:** 


### THE INSTITUTIONAL WORKSHOP.

### WITHIN THE HOSPITALS.

### THE ROYAL INFIRMARY, DUNDEE.

(By OUR SPECIAL COMMISSIONER.)

It was a brilliant day when we arrived at the doors of this institution. The elevation of the buildings is decidedly good, and the site is located upon a hill above the town, from which a fine view may be obtained on a clear day. This site has only one drawback, which it would have been difficult to avoid. When the wind is in the south-west all the smoke from the many works in the town below the hill passes directly over the infirmary buildings.

The rate of expenditure per occupied bed is always considerably less in the north than in the south. Thus the cost of each occupied bed at Dundee is under £58, as compared with £75 to £90 per bed at the same class of institution in London. In former years the cost per bed at Dundee has been even less than £58, a fact which caused a correspondent to send us a communication published in THE HOSPITAL of November 17th, 1888, to the effect that the Dundee Infirmary "resembled a workhouse rather than a hospital, and that interiorly it had a dreary, neglected, and repelling appearance."

It had not been possible for us, as we had hoped to do, to pay a visit to the Dundee Infirmary until a few weeks ago, when we were pleased to find that the hospital, though built upon the corridor system, contains wards which would compare favourably with those of any hospital built at the beginning of the present century, the date of Dundee Infirmary. Further, we were glad to find that, so far as appearance is concerned, they leave little or nothing to be desired. The corridors are spacious and airy; they act not only as passages of communication, but from them on the side, opposite to the wards, open out lavatories and bath-rooms. Of course, it would not be fair to criticise, from the modern point of view, the structural arrangements of an institution built nearly a hundred years ago. The lavatories and bath-rooms are not detached from the main building, nor are they separated from it by cross ventilation. The fittings, too, are old, though several of them have been, or are in course of being, replaced by appliances of a more modern type.

As erysipelas is not unknown in the wards, and a large number of typhoid and diphtheritic cases have arisen in the course of the year, the directors might wisely consider whether it would not be desirable to reconstruct the present lavatories and bath-rooms by placing them in towers at the back of the building, where they could be disconnected from the corridors. The money required to carry out this work would not be excessive, and sooner or later steps will no doubt be taken in the direction indicated.

The strictest economy is rightly enforced by the directors. There is evidence throughout the whole of the hospital that bit by bit money has been wisely spent upon improvements, and that there is a desire to make the hospital as efficient as possible. Having regard to the moderate expenditure at the date when the buildings were first erected, we found the Dundee Infirmary to be surprisingly efficient and satisfactory. The appearance of the wards, as we have said, was pleasing, bright, and cheerful; the corridors were spacious and bright, and both wards and corridors compare very favourably in appearance with those of the Western Infirmary, Glasgow. There can be no doubt that at the present time, at any rate, no one can justly say that any part of the hospital is dreary, neglected, or repelling in appearance. On the contrary, a careful inspection of the institution convinced us that in every department the arrangements were adequate, and the comfort of the staff was specially provided for. We are glad to have the opportunity of making this amend to the governing authorities of so important a medical institution, and we congratulate them upon the efficiency with which they maintain the Royal Infirmary, Dundee.

#### SPECIAL POINTS.

At the present time a nurses' home is being erected at the back of the hospital for the accommodation of ten nurses to form a private staff. These nurses will receive on engagement wages of £30 per annum, increasing to £36 per annum, and they will be entitled to a share of the profits made by their work. The institution employs at the present time thirty-six nurses, one third of whom are accommodated with single bed-rooms, and the rest in rooms containing two beds, which an inspection showed to be unusually large, bright, and pleasant. There is a separate dining-hall for the nurses, and also a comfortably furnished recreation-room containing a piano, library, &c., for the nurses' use. The accommodation for the servants is also good. The laundry and kitchens are well up to date. There is a lawn tennis ground for the use of the nurses, who appeared to be both happy and comfortable. Probationers are received for training, and are paid £12 the first year, £18 the second, and £21 the third. They continue their training until they become staff nurses, and receive £24 per annum. Staff nurses are paid £30 per annum, increasing to £36. All these facts go to prove that the present managers of the Dundee Royal Infirmary desire to keep up todate. We were convinced from all we saw that they ought to receive cordial recognition, from the success they have achieved, at the hands of their fellow townsmen. Dr. McCosh takes the greatest interest in his work. He does infinitely more of the routine and medical work than most hospital superintendents, and much of the institution's present efficiency must be placed to his credit. We have noticed Dr. McCosh's retirement in another place in to-day's issue.

